# Left ventricular function during porcine-resuscitated septic shock with pre-existing atherosclerosis

**DOI:** 10.1186/s40635-016-0089-y

**Published:** 2016-06-06

**Authors:** Benedikt L. Nußbaum, Oscar McCook, Clair Hartmann, José Matallo, Martin Wepler, Elena Antonucci, Miriam Kalbitz, Markus Huber-Lang, Michael Georgieff, Enrico Calzia, Peter Radermacher, Sebastian Hafner

**Affiliations:** Klinik für Anästhesiologie, Universitätsklinik Ulm, Ulm, Germany; Institut für Anästhesiologische Pathophysiologie und Verfahrensentwicklung, Universitätsklinik Ulm, Helmholtzstraße 8/1, 89081 Ulm, Germany; Department of Surgical Sciences and Integrated Diagnostics, IRCCS San Martino IST, University of Genova, Genova, Italy; Klinik für Unfall-, Hand-, Plastische- und Wiederherstellungschirurgie, Universitätsklinik Ulm, Ulm, Germany

**Keywords:** Septic shock, Septic cardiomyopathy, Left ventricular function, Ventricular dilatation, Atherosclerosis, Comorbidity, CSE, Nitrotyrosine

## Abstract

**Background:**

Reversible, depressed cardiac function is frequently encountered during septic shock and commonly called septic cardiomyopathy. Previous studies demonstrated reduced ejection fraction and left ventricular dilatation in both humans and animal models. However, the majority of the studies in humans excluded pre-existing cardiac disease and animal studies were performed on healthy specimen and/or without vasopressor support during sepsis. In order to more closely mimic the actual patients’ conditions on intensive care units and to assess the influence of both cardiac comorbidity and vasopressor support on septic cardiomyopathy, we evaluated the left ventricular function in a porcine model of resuscitated septic shock with pre-existing atherosclerosis.

**Methods:**

Hypercholesterolaemic, atherosclerotic pigs due to homozygous low-density lipoprotein receptor mutation and high-fat diet were anaesthetised and surgically instrumented. Faecal peritonitis was induced by inoculation of autologous faeces into the peritoneal cavity in *n* = 8 animals; *n* = 5 pigs underwent sham procedure. Sepsis resuscitation included administration of fluids and noradrenaline. Left ventricular function was analysed via pressure-conductance catheters before, 12 and 24 h after the induction of sepsis.

**Results:**

The main findings were impaired ventricular dilatation (no significant change in the left ventricular end-diastolic volume) and unchanged ejection fraction in septic pigs with pre-existing atherosclerosis. The relaxation time constant *τ* decreased while dp/dt_max_ increased. Cardiac nitrotyrosine formation increased while expression of the endogenous hydrogen sulphide (H_2_S)-producing enzyme cystathionine γ-lyase (CSE) decreased.

**Conclusions:**

The data of the present study are in conflict with previously published data from healthy animal models, most likely as a result of ongoing resuscitation including noradrenaline treatment or intrinsic pathophysiologic processes of the pre-existing atherosclerosis. Moreover, increased nitrotyrosine formation and decreased expression of CSE suggest the implication of augmented oxidative/nitrosative stress and/or reduced bioavailability of nitric oxide as well as diminished endogenous H_2_S release in the pathophysiology of septic cardiomyopathy.

**Electronic supplementary material:**

The online version of this article (doi:10.1186/s40635-016-0089-y) contains supplementary material, which is available to authorized users.

## Background

There is extensive evidence for reversible intrinsic cardiac dysfunction and myocardial depression in patients with sepsis or septic shock, a condition frequently called septic cardiomyopathy [1]. These cardiac abnormalities can affect the left as well as the right ventricle and may interfere with both the systolic and the diastolic properties of the heart [2] with most of the studies focussing on the left ventricular (LV) systolic function. Parker et al. described a reversible reduction in LV function as assessed by ejection fraction (EF) in patients with septic shock. Of note, compared to non-survivors, survivors of sepsis presented with a lower EF and a marked LV dilatation. The authors suggested that LV dilatation might be an adaptive response of the heart to maintain stroke volume despite sepsis-induced systolic cardiac depression [3]. Subsequent studies confirmed LV dilatation in sepsis in animal models [4, 5] and humans [6]. Nevertheless, the hypothesis of adaptive ventricular dilatation remains controversial. Several more recent studies using echocardiography did not find LV dilatation in septic patients [7, 8]. Moreover, the majority of clinical studies in humans evaluating cardiac function in sepsis did not consider the patients’ comorbidities. Experimental studies also used only healthy animal models without underlying (cardiac) comorbidity. However, a high proportion of patients with sepsis present with relevant comorbidities [9]. These comorbidities significantly influence sepsis-related mortality [10]. Besides, patients in septic shock on ICUs frequently need haemodynamic support with catecholamines, whereas the majority of the animal studies did not include vasopressors in their treatment protocols. Therefore, to appreciate the importance of both the frequently present pre-existing (cardiac) comorbidity and vasopressor support, we studied cardiac function in a resuscitated porcine model of septic shock with pre-existing atherosclerosis.

## Methods

### Animals

The study was approved by the University of Ulm Animal Care Committee and the Federal Authorities for Animal Research. The experiments were performed in adherence to the National Institute of Health Guidelines on the Use of Laboratory Animals and the European Union “Directive 2010/63/EU on the protection of animals used for scientific purposes”. The present study is a post hoc analysis of the data available from the vehicle-treated group of a previous study [11] and sham-operated animals studied simultaneously. Thirteen castrated male familial hypercholesterolemia Bretoncelles Meishan (FBM) pigs with a median weight of 69 kg (interquartile range 65 to 73 kg) aged between 15 and 30 months were used. This pig strain is characterised by a homozygous low-density lipoprotein (LDL) receptor mutation and develops marked atherosclerosis under atherogenic diet [12, 13]. The pigs were fed with atherogenic diet (1 kg daily, 1.5 % cholesterol, 20 % bacon fat) for at least 9 months prior to the experiments. The phenotype has been characterised previously [14]. Briefly, FMB pigs on atherogenic diet exhibit significantly higher cholesterol levels compared to healthy German landrace swine of the same age. Plasma levels of 8-isoprostane are significantly increased, plasma nitrite/nitrate levels are significantly lower and creatinine clearance is also significantly reduced in contrast to healthy German landrace swine (Additional file 1) [14]. FBM pigs are further characterised by altered glucose homeostasis [11], and they present with modified receptor expression patterns (lower erythropoietin receptor expression as well as lower PPAR-β/δ expression compared to young healthy German landrace swine) [11, 14]. Moreover, despite similar organ dysfunction, FBM pigs displayed a different response to kidney ischemia/reperfusion injury with respect to nitrite/nitrate and 8-isoprostane levels (Additional file 2) [14, 15]. Histological examination of coronary arteries of the FBM pigs on high-fat diet confirmed classical features of atherosclerosis, such as asymmetric lesion formation, pathological intimal and medial thickening, lipid accumulation and foam cell formation (Additional files 3, 4, 5 and 6). The pathological altered intima was strongly positive for adipophilin, a marker for lipid accumulation [16], whereas unaffected areas of the intima were negative for adipophilin (Additional file 7). Adipophilin is suggested to be involved in atherogenesis, as it is induced by oxidized LDL in macrophages [17] and further contributes to lipid accumulation [18].

### Anaesthesia

Before the experiments, pigs were fasted for 12 h with free access to water. Intramuscular premedication consisted of 2.5 mg atropine and 5 mg kg^−1^ azaperone. After establishment of an intravenous access via the ear vein, anaesthesia was induced with propofol (1–2 mg kg^−1^) and ketamine (1–2 mg kg^−1^). The pigs were endotracheally intubated, and their lungs were mechanically ventilated (tidal volume 8 ml/kg, respiratory rate 8–12 adapted to achieve an arterial partial pressure of carbon dioxide (pCO_2_) of 35–45 mmHg, inspiratory/expiratory (I/E) ratio 1:1.5, fraction of inspiratory oxygen (FiO_2_) 35 %, positive end-expiratory pressure (PEEP) 10 cm H_2_O, peak airway pressure ≤40 mmHg). Anaesthesia was maintained with continuous intravenous infusion of pentobarbitone (8–12 mg kg^−1^ h^−1^). Buprenorphine was used for analgesia (30 μg kg^−1^ initially, further 10 μg kg^−1^ every 8 h as well as prior to surgery and induction of faecal peritonitis). Pancuronium (0.1 mg kg^−1^ h^−1^) ensured appropriate muscle relaxation. Ringer’s solution (10 ml kg^−1^ h^−1^) was infused for fluid homeostasis.

### Surgical procedures

Both internal jugular veins were exposed. A heat exchange catheter was inserted in the left internal jugular vein to control and maintain body core temperature at 37.5–38.5 °C. A central venous catheter sheath was placed in the right internal jugular vein. The central venous catheter was subsequently used for infusion therapy and application of intravenous drugs including catecholamines. A balloon-tipped thermodilution pulmonary artery catheter was inserted via the sheath and used for the measurement of the central venous pressure (CVP), mean pulmonary arterial pressure (MPAP), pulmonary artery occlusion pressure (PAOP) and cardiac output (CO). An arterial catheter was inserted into the femoral artery to monitor the arterial blood pressure (MAP). Exposure of the left carotid artery was followed by the insertion of an arterial catheter sheath for the introduction of a pressure-conductance catheter allowing the analysis of LV function. A midline mini-laparotomy allowed the insertion of a catheter into the bladder to collect urine. Two tubes were placed through the abdominal wall into the peritoneal cavity for subsequent induction of peritonitis. During surgery, hydroxyethyl starch was infused as needed to maintain cardiac filling pressures.

### Experimental protocol

After 4 h of surgery, the pigs were allowed to recover for 8 h before baseline data were collected. Subsequently, faecal peritonitis was induced. For this purpose, 1 g kg^−1^ autologous faeces was collected during premedication, dissolved in 500 ml 0.9 % saline and incubated at 38 °C for 12 h. Of the supernatant, 3 ml kg^−1^ was injected into the peritoneal cavity via the abdominal tubes. Eight animals received autologous faeces while five animals underwent sham surgery without inoculation of faeces. Animals were monitored for 24 h, and additional data sets were acquired 12 and 24 h after the induction of peritonitis. Ringer’s solution (10 ml kg^−1^ h^−1^) was continuously infused. For additional circulatory support, hydroxyethyl starch (10 or 5 ml kg^−1^ h^−1^ if PAOP or CVP >18 mmHg, respectively) was administered. If MAP remained below baseline values despite volume resuscitation, noradrenaline was used to stabilize MAP at pre-peritonitis values. However, infusion rates of noradrenaline were not further increased if the heart rate was higher than 170 beats per minute (bpm) in order to avoid tachycardia-induced myocardial ischemia. One animal in the sepsis group had a heart rate of 173 bpm after 24 h, and therefore, noradrenaline application was not further increased. Respirator settings were modified (I/E ratio 1:1, PEEP 12 or 15 cm H_2_O) during the experiments when the Horowitz index (ratio of arterial oxygen partial pressure (PaO_2_) to FiO_2_) dropped below 300 or 200 mmHg, respectively. FiO_2_ was incrementally increased to maintain an arterial haemoglobin O_2_ saturation of ≥90 %. At the end of the experiment, the pigs were euthanized under deep anaesthesia via injection of potassium chloride.

### Measurements

Immediately before, 12 and 24 h after induction of faecal peritonitis, data sets were collected. Measurements included haemodynamics (MAP, MPAP, PAOP, CVP, heart rate, CO), arterial and mixed venous blood gases (pO_2_, pCO_2_), glucose, lactate, base excess and LV function. For the assessment of LV function, a pressure-volume catheter (CD Leycom, Hengelo, The Netherlands) was placed via the arterial catheter sheath in the left carotid artery. Subsequently, the catheter was advanced towards the heart and into the left ventricle across the aortic valve under the control of the online arterial pressure signal. The catheter was connected to a Sigma 5 DF signal processor (CD Leycom). Prior to insertion, the catheter was put into 0.9 % saline at room temperature and calibrated according to the manufacturer’s protocol. The principle of the pressure-volume catheter has been described previously [19]. Briefly, the catheter possesses a tip sensor allowing the measurement of the left ventricular pressure. Additionally, 12 electrodes are positioned along the catheter. The four outermost electrodes generate a small electric field. The remaining inner electrodes measure segmental voltage gradients during the cardiac cycle. Segmental conductance values are calculated from these voltage signals measured along the catheter. Each conductance signal represents a segmental volume of the ventricle. However, the conductance-derived volumes differ from the true cardiac volumes, as the measured conductance is not solely influenced by ventricular volumes but also by structures surrounding the ventricular cavity, e.g. the myocardium. This part of the conductance is called the *parallel conductance* and can be determined by the injection of a bolus of hypertonic (20 %) saline via the central venous catheter. The saline bolus is assumed to temporarily change the blood conductivity without affecting the parallel conductance. From this change in conductivity, parallel conductance can be obtained. Knowing the specific conductance of blood, the electrode spacing and the parallel conductance, the total volume of the ventricle can finally be calculated. Thus, the pressure-volume catheter allows continuous online recording of cardiac pressures and volumes. Further analysis of cardiac data from pressure volume loops was performed using Conduct NT software (CD Leycom).

### Immunohistochemistry

Heart specimens were immediately collected post-mortem and analysed for nitrotyrosine formation and expression of the endogenous H_2_S-producing enzyme cystathionine γ-lyase (CSE). Therefore, the specimen was fixed in formalin, embedded in paraffin, dewaxed in xylene and rehydrated with a graded series of ethanol. After incubation in citrate buffer and boiling for heat-induced antigen retrieval, samples were blocked with goat sera and subsequently incubated with primary anti-nitrotyrosine (Millipore, Schwalbach, Germany) or anti-CSE antibodies (Abnova, Taipei City, Taiwan). Primary antibody detection was performed by alkaline phosphatase-conjugated secondary antibodies and visualized with a red chromogen (Dako APAAP REAL; Dako, Hamburg, Germany) followed by counterstaining with haematoxylin. Slides were visualized using a Zeiss Axio Imager A1 microscope with a ×10 objective. Quantification for intensity was performed using the AxioVision 4.8 software (Zeiss, Jena, Germany). Results are presented as median densitometric sum red. Specimens of coronary arteries were stained with haematoxylin. Polyclonal anti-adipophilin antibody (Progen, Heidelberg, Germany) was used for adipophilin staining.

### Statistical analysis

All data are expressed as median (IQ range). Differences within each group were tested by using a Friedman analysis of variance on ranks and a subsequent Dunn’s test for multiple comparisons with Bonferroni correction. Inter-group differences were analysed by the Mann-Whitney rank sum test. A *p* value less than 0.05 was considered statistically significant. GraphPad Prism 6 software was used for statistical evaluation and graphical display.

## Results

Table 1 summarises the haemodynamic changes. MAP was significantly decreased in septic animals after 24 h compared to sham-operated pigs (*p* < 0.05) despite aggressive supportive volume and vasopressor therapy. Consequently, heart rate (*p* < 0.005) and cardiac output (*p* < 0.005) were significantly increased at 12 and 24 h of sepsis when compared to pre-shock values. MPAP progressively increased both in septic and sham animals but was significantly higher in septic animals at the end of the experiment (*p* < 0.02). Systemic vascular resistance significantly decreased in septic animals compared to baseline (*p* < 0.003) and to control group (*p* < 0.005).

**Table 1:** Hemodynamic data

	Baseline	12 h	24 h
MAP (mmHg)			
Sham	100 (90; 106)	95 (89; 97)	103 (94; 119)
Sepsis	103 (91; 112)	94 (89; 102)^*^	71 (62; 101)^*, **^
MPAP (mmHg)			
Sham	22 (20; 25)	26 (24; 28)	28 (24; 31)^*^
Sepsis	23 (20; 26)	31 (25; 41)^*^	39 (32; 40)^*,**^
PAOP (mmHg)			
Sham	9 (7; 16)	10 (9; 15)	11 (7; 16)
Sepsis	11 (6; 13)	15 (9; 15)	18 (16; 20)^*,**^
CVP (mmHg)			
Sham	8 (7; 13)	9 (8; 14)	10 (9; 17)^*^
Sepsis	10 (6; 13)	14 (9; 15)	17 (14; 18)^*^
HR (beats/min)			
Sham	88 (73; 104)	90 (82; 105)	102 (68; 115)
Sepsis	88 (74; 106)	143 (114; 159)^*,**^	156 (140; 166)^*,**^
Cardiac output (l/min)			
Sham	3.9 (3.4; 6.2)	4.1 (3.7; 6.6)	4.4 (2.9; 6.5)
Sepsis	4.5 (3.5; 5.4)	6.8 (5.8; 8.9)^*^	6.3 (4.6; 10.8)^*^
SVR (dyn^*^s/cm^5^)			
Sham	1870 (1195; 2002)	1636 (1075; 1861)	1883 (1243; 2237)
Sepsis	1734 (1515; 1833)	961 (638; 1245)^**^	806 (560; 917)^*,**^

All data are medians (25; 75 percentile). Sham *n* = 5; sepsis *n* = 8

*MAP* mean arterial pressure, *MPAP* mean pulmonary artery pressure, *PAOP* pulmonary artery occlusion pressure, *CVP* central venous pressure, *HR* heart rate, *SVR* systemic vascular resistance

^*^ *p* < 0.05 compared to baseline; ^**^ *p* < 0.05 compared to sham group

Table 2 demonstrates the acid-base and gas exchange data. Septic shock was associated with deteriorated pulmonary gas exchange resulting in a significant fall of PaO_2_ (*p* < 0.05) and the Horowitz index (*p* < 0.001) as well as progressive lactic acidosis.

**Table 2:** Gas exchange, acid-base balance and glucose

	Baseline	12 h	24 h
Arterial pH			
Sham	7.46 (7.44; 7.46)	7.43 (7.42; 7.45)	7.44 (7.44; 7.46)
Sepsis	7.45 (7.43; 7.48)	7.46 (7.40; 7.48)	7.35 (7.19; 7.42)^*,**^
PaCO_2_ (mmHg)			
Sham	35 (35; 39)	37 (34; 38)	35 (33; 36)
Sepsis	38 (34; 40)	38 (33; 40)	38 (33; 42)
PaO_2_ (mmHg)			
Sham	158 (142; 180)	160 (144; 185)	159 (138; 178)
Sepsis	170 (161; 183)	155 (129; 163)	110 (71; 144)^*,**^
Horowitz index (mmHg)			
Sham	495 (429; 536)	484 (436; 552)	484 (425; 528)
Sepsis	550 (489; 574)	477 (382; 505)	225 (75; 388)^*,**^
Glucose (mg/dl)			
Sham	128 (114; 141)	74 (69; 131)^*^	77 (72; 125)
Sepsis	115 (89; 119)	76 (65; 96)^*^	87 (72; 123)
Lactate (mmol/l)			
Sham	1.4 (1.0; 1.6)	0.6 (0.4; 1.0)	0.6 (0.6; 1.2)
Sepsis	0.8 (0.6; 1.5)	1.0 (0.7; 1.7)	4.3 (1.9; 8.6)^*, **^
Base excess (mmol/l)			
Sham	1.1 (0.8; 1.8)	-0.3 (-0.7; 0.7)^*^	-0.1 (-1.5; 0.7)
Sepsis	1.5 (0.4; 2.3)	1.7 (-0.7; 4.6)	-5.0 (-12.0; -2.9)^*, **^

All data are medians (25; 75 percentile). Sham *n* = 5, sepsis *n* = 8

^*^ *p* < 0.05 compared to baseline; ^**^ *p* < 0.05 compared to sham group

The cardiac function data are summarized in Table 3. In line with a decreasing MAP, left ventricular end-systolic pressure (LVESP) was lower at the end of the experiment in septic pigs compared to pre-shock values. dp/dt_max_ values significantly increased after 12 and 24 h in septic pigs compared to baseline values. dp/dt_min_, stroke volume, ejection fraction, left ventricular end-systolic volume (LVESV) and left ventricular end-diastolic volume (LVEDV) did not reveal significant statistical differences between both groups and within groups compared to baseline. However, slightly lower LVEDV together with higher left ventricular end-diastolic pressure (LVEDP) after 24 h of sepsis indicate increased LV stiffness. The isovolumetric relaxation time constant *τ* significantly decreased in septic pigs compared to control pigs (*p* < 0.02) and to baseline values (*p* < 0.01).

**Table 3:** Cardiac data

	Baseline	12 h	24 h
LVESP (mmHg)			
Sham	102 (92; 117)	99 (91; 114)	112 (87; 123)
Sepsis	105 (86; 114)	100 (89; 110)	90; 67; 101)^*^
LVEDP (mmHg)			
Sham	14 (9; 19)	14 (9; 20)	18 (13; 23)
Sepsis	11 (9; 17)	17 (14; 20)	18 (14; 21)
dp/dt_max_ (mmHg/s)			
Sham	1797 (1675; 2170)	2573 (1412; 4028)	2224 (1857; 3590)
Sepsis	1571 (1341; 2025)	4239 (1817; 4608)^*^	3150 (1794; 5122)^*^
dp/dt_min_ (mmHg/s)			
Sham	−2222 (−2473; −1904)	−2148 (−2423; −1293)	−2461 (−2748; −1246)
Sepsis	−1971 (−2257; −1735)	−2090 (−2436; −1743)	−2180 (−2719; −1556)
SV (ml)			
Sham	50 (46; 59)	47 (45; 59)	47 (43; 54)
Sepsis	44 (39; 67)	49 (39; 67)	42 (31; 66)
EF (%)			
Sham	45 (44; 49)	49 (44; 66)	39 (34; 49)
Sepsis	50 (41; 61)	49 (34; 58)	60 (40; 78)
LVESV (ml)			
Sham	59 (52; 65)	49 (24; 75)	75 (46; 104)
Sepsis	37 (30; 80)	53 (41; 109)	31 (24; 44)
LVEDV (ml)			
Sham	116 (102; 129)	105 (78; 137)	131 (97; 168)
Sepsis	94 (78; 118)	99 (90; 204)	86 (62; 114)
Tau (ms)			
Sham	28 (26; 35)	27 (25; 41)	26 (23; 45)
Sepsis	28 (25; 38)	21 (19; 26)	18 (14; 23)^*,**^

All data are medians (25; 75 percentile). Sham *n* = 5; sepsis *n* = 8

*LVESP* left ventricular end-systolic pressure, *LVEDP* left ventricular end-diastolic pressure, *dp/dt*_*max*_ maximal rate of pressure increase, *dp/dt*_*min*_ maximal rate of pressure decrease, *SV* stroke volume, *EF* ejection fraction, *LVESV* left ventricular end-systolic volume, *LVEDV* left ventricular end-diastolic volume

^*^ *p* < 0.05 compared to baseline; ^**^ *p* < 0.05 compared to sham group

**Fig. 1:**
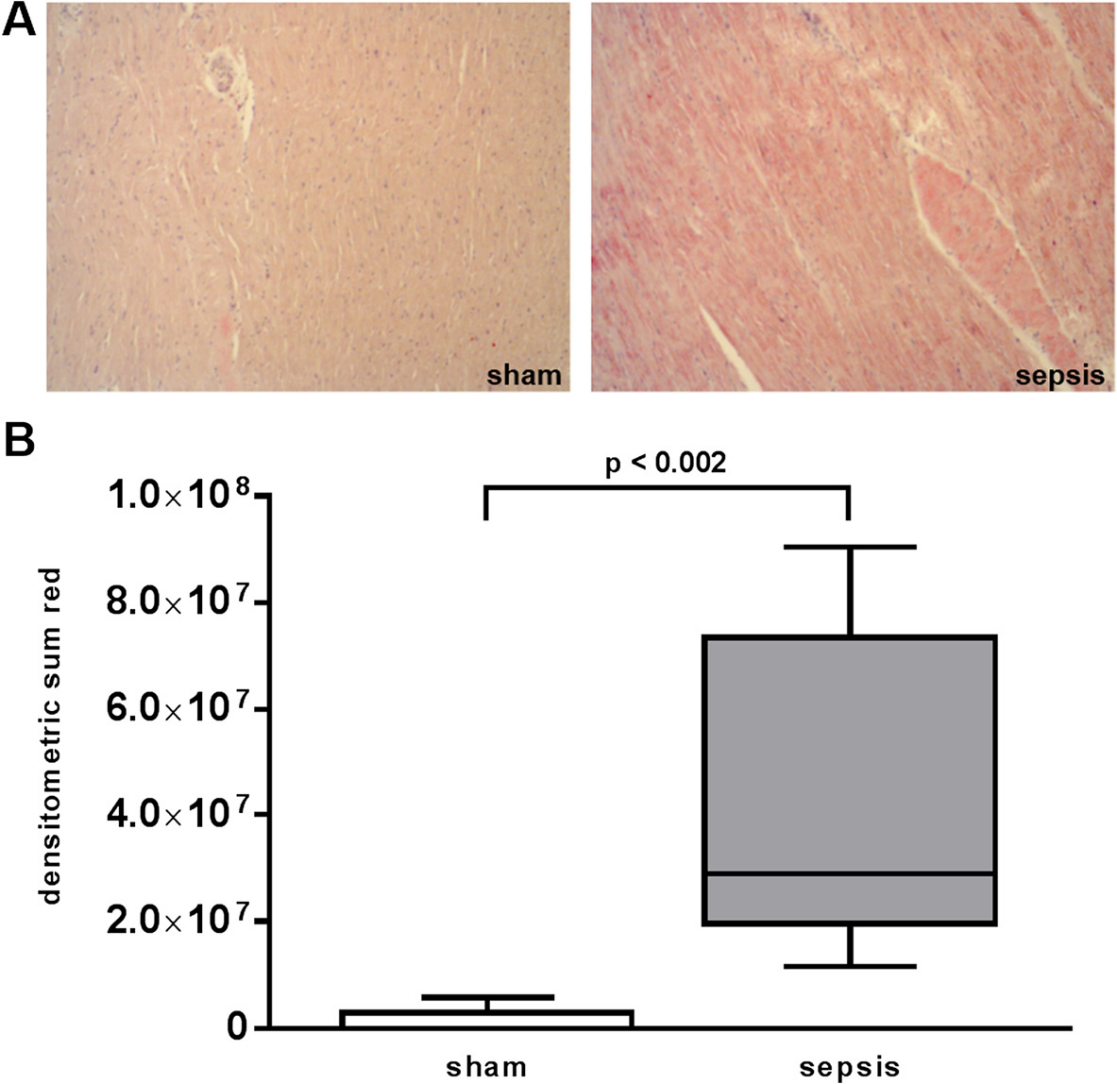
Nitrotyrosine staining of heart specimen collected at the end of the experiment after 24 h. **a** Two representative histological images of sham (*left*) and septic (*right*) animals (×10 magnification). **b** Quantitative results of densitometric analysis. Data are presented as median (range). Sham *n* = 5, sepsis *n* = 8 animals

**Fig. 2:**
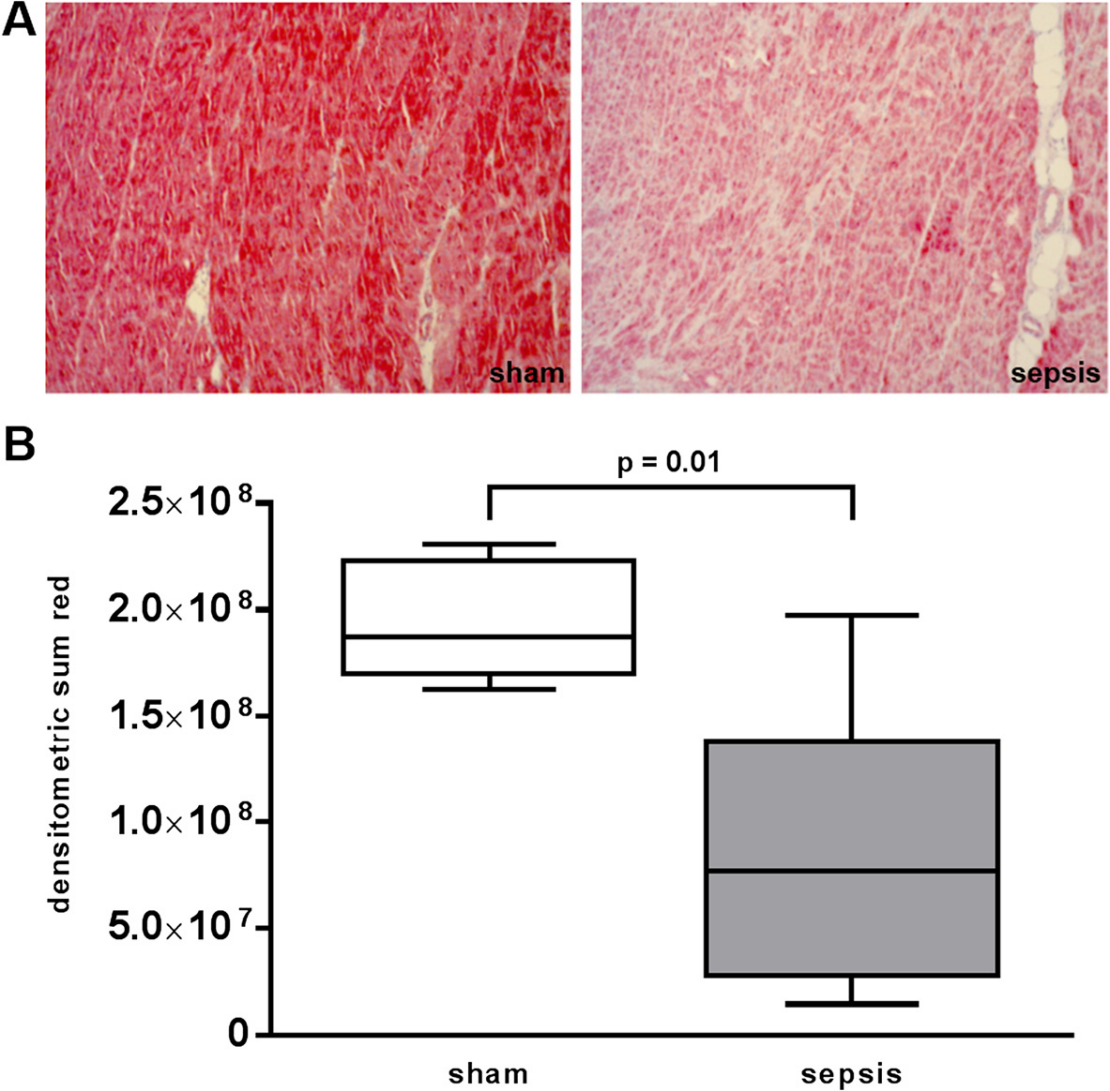
CSE (cystathionine γ-lyase) staining of the heart specimen collected at the end of the experiment after 24 h. **a** Two representative histological images of sham (*left*) and septic (*right*) animals (×10 magnification). **b** Quantitative results of densitometric analysis. Data are presented as median (range). Sham *n* = 5, sepsis *n* = 8 animals

Sepsis significantly increased cardiac nitrotyrosine formation (*p* < 0.002, Fig. 1). In contrast, expression of the endogenous H_2_S-producing enzyme CSE significantly decreased during sepsis (*p* = 0.01, Fig. 2).

## Discussion

**Fig. 3:**
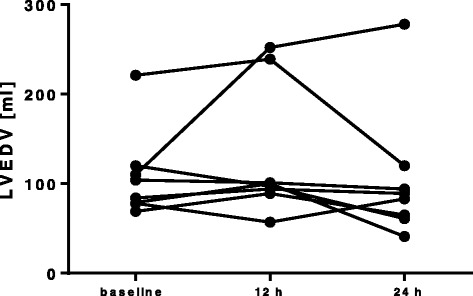
Individual time course of the left ventricular end-diastolic volume (LVEDV) of septic animals

The aim of the present study was the evaluation of the left ventricular function in a porcine model of resuscitated septic shock with pre-existing atherosclerosis. The main findings were (1) impaired LV dilatation (no significant change in LVEDV), (2) unchanged ejection fraction and (3) increased cardiac nitrotyrosine formation and reduced cardiac expression of CSE in septic animals with pre-existing atherosclerosis and ongoing vasopressor support. Parker et al. initially described a reversible reduction of LV systolic function measured as EF and a marked LV dilatation in patients with septic shock. Interestingly, these changes only occurred in survivors of sepsis while non-survivors showed no ventricular dilatation and presented with preserved EF. However, afterload assessed as systemic vascular resistance index was decreased in non-survivors, which might have resulted in sustained EF despite possible systolic impairment. LV dilatation was considered as adaptive response to maintain stroke volume despite cardiac dysfunction [3]. Subsequently, LV dilatation was also demonstrated in canine [4] and murine septic shock [5]. Of note, only a proportion of mice (37 %) underwent ventricular dilatation with improved survival. More recent studies in humans also found LV dilatation, even though less consistent: Bouhemad et al. described LV dilatation in a subgroup of patients with systolic dysfunction [6]. Other studies reported significantly larger LVEDVs in survivors of sepsis compared to non-survivors [20–22]. In contrast to the aforementioned studies, the concept of ventricular dilatation was challenged by other authors. Although confirming frequent systolic dysfunction in sepsis, no change in LV volumes was observed [7, 8, 23]. A recent meta-analysis provided significant evidence for non-indexed LV dimensions being larger in survivors of severe sepsis and septic shock [24]. Several issues need to be addressed to account for the conflicting results. First, different methods were used for cardiac assessment comprising radionuclide cineangiography, thermodilution technique and transthoracic and transesophageal echocardiography. However, invasive and non-invasive techniques may yield different results [25]. Furthermore, as septic cardiomyopathy is a reversible process [26]; serial investigations are required to capture the dynamic process of LV dilatation. In other words, the timing of cardiac evaluation plays a crucial role. Depending on the time of the first assessment, incidence of LV dysfunction ranged from 18 to 65 % [1]. Moreover, as LV dilatation commonly does not occur in all patients, LV dimensions have to be addressed for each individual patient. In fact, one individual pig displayed an increase in LVEDV (110 ml before sepsis, 278 ml after 24 h), while LVEDV remained stable or even decreased within 24 h in all other animals in our study (Fig. 3).

In our study on hypercholesterolaemic pigs with pre-existing atherosclerosis, dp/dt_max_ was significantly elevated in septic animals after 24 h. The increase in dp/dt_max_ most likely reflects catecholamine treatment and the positive inotropic effects of noradrenaline [27] (median dose of noradrenaline 0.82 μg kg^−1^ min^−1^ (0.52–2.11) in septic versus 0.06 μg kg^−1^ min^−1^ (0.02–0.13) in sham pigs). As dp/dt_max_ is essentially heart rate dependent [28], the sepsis- and noradrenaline-induced tachycardia further contributed to the raise of dp/dt_max_. In contrast to dp/dt_max_, EF remained largely unchanged for 24 h in the septic swine. However, EF represents a load-dependent parameter of LV function [29], and the significant reduction in systemic vascular resistance after 12 and 24 h of sepsis as well as the high doses of noradrenaline likely contributed to the sustained EF. It is therefore conceivable that the load as well as the heart rate dependence of dp/dt_max_ and EF in combination with NA treatment may have obscured the detection of impaired LV systolic function. Of note, in a rat model of septic shock with severe myocardial dysfunction, noradrenaline administration 18 h after the induction of sepsis significantly improved cardiac performance associated with increased EF and dp/dt_max_ [30].

The heart-rate-independent relaxation time constant *τ* was significantly decreased in septic animals at 24 h. The decline of *τ* can also be attributed to noradrenaline treatment, as the positive lusitropic effect of catecholamines enables faster diastolic relaxation [27]. In contrast to our results in a fluid- and noradrenaline-resuscitated model of septic shock, dp/dt_max_ declined and *τ* increased in septic mice [31] and endotoxemic pigs [32] without vasopressor support. Although LVEDV and LVEDP did not significantly change in septic animals, a trend towards increased LVEDP could be observed, which is in line with a significantly elevated filling pressure (PAOP). Together with a stable or even slightly reduced LVEDV, the findings on diastolic function suggest a reduction in LV compliance. This rationale is underscored by a study in patients with sepsis reporting significantly lower indexed LVEDV in patients with coronary artery disease (CAD) compared to patients without CAD: the authors hypothesized that the pre-existing atherosclerosis contributed to the impairment of ventricular dilatation [33]. Furthermore, a study in 40 patients including 12 patients with pre-existing cardiac disease reported that 7 patients developed reversible cardiac dysfunction during sepsis with reduced EF and larger LV end-diastolic diameter compared to patients with normal EF. Interestingly, none of these 7 patients had pre-existing heart disease, and despite an overall mortality of 23 %, all these patients survived [34]. Injection of endotoxin in volunteers without comorbidities resulted in reduced ventricular performance and increased LVEDV index [35].

The underlying mechanisms of septic cardiomyopathy are still a matter of debate [36]. In our study, sepsis significantly increased cardiac nitrotyrosine formation. Augmented nitrotyrosine formation has also been demonstrated in the heart specimen of septic patients [37, 38]. Nitrotyrosine is a well-established marker for both augmented oxidative and nitrosative stress resulting from the nitration of protein tyrosine residues by peroxynitrite, a product of the reaction of nitric oxide (NO) with superoxide [39]. Therefore, increased nitrotyrosine formation may be associated with reduced bioavailability of NO [40], and both elevated nitrotyrosine as well as low levels of NO are implicated in the pathophysiology of atherosclerosis [41, 42]. However, NO improves diastolic relaxation and promotes LV distension [43, 44]. Thus, augmented oxidative as well as nitrosative stress itself and/or the reduced availability of NO as a consequence of increased nitrotyrosine formation might be a possible explanation for the lack of LV dilatation.

Additionally, sepsis significantly reduced cardiac expression of CSE, an endogenous hydrogen sulphide (H_2_S)-producing enzyme. CSE and endogenous H_2_S have also been implicated in atherogenesis: Overexpression of CSE exerted anti-atherosclerotic effects [45], whereas CSE knockout promoted disease progression in a murine model of diet-induced atherosclerosis [46]. Plasma levels of H_2_S have been shown to be significantly reduced in patients with CAD [47]. Moreover, CSE expression was reduced in a murine model of heart failure [48], and CSE activity was diminished in a model of myocardial ischemic injury [49]. Administration of the H_2_S donor sodium hydrosulphide (NaHS) improved diastolic heart function by significantly reducing LVEDP and improving dp/dt_min_ in isolated rat hearts subjected to I/R [50] as well as in vivo [49]. Thus, considering LV dilatation as a protective, adaptive response in sepsis [51], reduced availability of H_2_S due to decreased expression of CSE in the atherosclerotic pigs in our study might also be responsible for impaired diastolic cardiac function besides increased nitrotyrosine formation.

### Limitations

The major limitation of our study represents the lack of healthy control pigs. As mentioned above, the present study is a post hoc analysis of the data available from the vehicle-treated group of a previous study [11] and sham-operated animals studied simultaneously. Hence, we cannot differentiate between the effects ICU procedures and ongoing noradrenaline treatment or the underlying atherosclerosis. Determinants of systolic LV function assessed in the present study did not reflect septic myocardial dysfunction. However, these parameters are strongly influenced by the positive inotropic effects of noradrenaline [27]. Therefore, it is possible that the high doses of vasopressor support in the septic pigs might have obscured the detection of systolic LV impairment. The rationale for the inclusion of continuous noradrenaline infusion in the therapeutic regimen was (i) to closely mimic the patients’ conditions on ICUs and (ii) to avoid hypotension-induced coronary hypoperfusion with subsequent cardiac depression due to ischemia.

In the initial report by Parker et al., only survivors of sepsis displayed the dilatational response of the left ventricle, whereas LVEDV of non-survivors remained largely unchanged. As the experiments of the present study were terminated 24 h after the induction of peritonitis, sepsis mortality could not be assessed. Therefore, we cannot exclude the possibility that the severity of our sepsis model might have impaired possible LV dilation in line with other human non-survivors data [20, 21]. However, septic patients with CAD displayed lower indexed LVEDV compared to patients without CAD, and albeit mortality in both groups did not differ, the severity of sepsis most likely was much less pronounced than in the present study: no vasopressor treatment was required [33]. Furthermore, the dilatational response of the left ventricle in sepsis commonly occurs only in a proportion of patients, e.g. 37 % in a mouse study [5] or 10 from 20 patients in the initial report by Parker et al. [3]. Hence, the relatively small number of *n* = 8 septic animals in our study might have led to the underestimation of possible dilatory effects in the atherosclerotic animals. However—considering the percentages of dilatation in the aforementioned studies—one would expect at least more than only one dilator described here. It is conceivable that pre-existing CAD does not completely prevent LV dilatation but rather reduce its incidence. Clearly, further studies with larger groups are needed to draw valid conclusions on the frequency of the occurrence of LV adaption. The duration of our study was only 24 h. We cannot exclude the possibility that LV dilatation might have occurred at later time points. In awake dogs with sepsis [4] and in chronic porcine endotoxemia [52, 53], LV dilatation was reported after 48 h. However, in the initial Parker study, myocardial dysfunction was present already during the first assessment within 24 h and LVEDV was highest at that time point, and subsequently decreased within the next 7–10 days. Moreover, we detected significantly increased LVEDV in murine model of sepsis after 21 and 24 h [44]. Therefore, we considered the short-term model of 24 h adequate to detect LV alterations due to sepsis. Nevertheless, longer experiments in the future could even better help to evaluate the dynamic process of septic cardiomyopathy.

## Conclusions

We evaluated cardiac function during resuscitated septic shock in a porcine model of pre-existing atherosclerosis. Neither decreased ejection fraction nor adaptive LV dilatation could be detected. Increased formation of nitrotyrosine and reduced expression of CSE suggest augmented oxidative/nitrosative stress and/or reduced bioavailability of NO or H_2_S to be involved in the impairment of LV distension in septic cardiomyopathy. As previous studies describing LV dilatation mainly used healthy animal models without vasopressor therapy or excluded cardiac comorbidities in humans, ongoing catecholamine therapy or intrinsic pathophysiologic processes of atherosclerosis are most likely responsible for the opposing results.

## Abbreviations

BPM, beats per minute; CAD, coronary artery disease; CO, cardiac output; CSE, cystathionine γ-lyase; CVP, central venous pressure; EF, ejection fraction; FBM, familial hypercholesterolemia Bretoncelles Meishan; FiO_2_, fraction of inspiratory oxygen; H_2_S, hydrogen sulphide; I/E, inspiratory/expiratory; ICU, intensive care unit; LV, left ventricular; LVEDP, left ventricular end-diastolic pressure; LVEDV, left ventricular end-diastolic volume; MAP, mean arterial pressure; MPAP, mean pulmonary arterial pressure; NO, nitric oxide; PAOP, pulmonary artery occlusion pressure; PEEP, positive end-expiratory pressure

## Additional files

Additional file 1: Phenotype of familial hypercholesterolemia Bretoncelles Meishan (FBM) pigs. Phenotype of familial hypercholesterolemia Bretoncelles Meishan (FBM) pigs with atherogenic diet for at least 9 months compared to healthy German landrace swine. *n* = 20 for FBM, *n* = 15 for landrace for cholesterol; *n* = 19 each for creatinine clearance, nitrite/nitrate and 8-isoprostane. Data are median (range) or mean ± standard deviation [14]. (DOCX 55 kb) Additional file 2: Response to ischemia/reperfusion injury of familial hypercholesterolemia Bretoncelles Meishan (FBM) pigs. Response to kidney ischemia/reperfusion injury with comparable post-ischemic organ dysfunction of familial hypercholesterolemia Bretoncelles Meishan (FBM) pigs with atherogenic diet for at least 9 months compared to healthy German landrace swine. *n* = 7 for FBM, *n* = 10 for landrace. Data are median (range) [14, 15]. (DOCX 14 kb) Additional file 3: Histology of coronary artery of familial hypercholesterolemia Bretoncelles Meishan (FBM) pig. Haematoxylin staining of the left coronary artery of a FBM pig on atherogenic diet. A. 2.5-fold magnification demonstrating pronounced pathological intimal thickening with narrowing of the arterial lumen. B–D. Tenfold magnification of different areas of the vessel. Asterisk (*) indicates areas of strong lipid accumulation. Red arrows mark the internal elastic lamina (single arrow) and the outer border of the media (double arrow). (JPG 140 kb) Additional file 4: Histology of coronary artery of familial hypercholesterolemia Bretoncelles Meishan (FBM) pig. Haematoxylin staining of coronary artery of a FBM pig on atherogenic diet. A 2.5- and B tenfold magnification demonstrating asymmetric atherosclerotic alterations with an instable plaque containing a large lipid core and associated thrombus formation. (JPG 52 kb) Additional file 5: Histology of coronary artery of familial hypercholesterolemia Bretoncelles Meishan (FBM) pig. Haematoxylin staining of right coronary artery of a FBM pig on atherogenic diet showing intimal thickening with marked lipid accumulation. ×40 magnification. (JPG 345 kb) Additional file 6: Histology of coronary artery of familial hypercholesterolemia Bretoncelles Meishan (FBM) pig. Haematoxylin staining of coronary artery of a FBM pig on atherogenic diet demonstrating asymmetric atherosclerotic lesion formation with luminal narrowing. Note the coincident medial thickening at sites of intimal proliferation in contrast to unaffected regions of the vessel. Magnification of 2.5-fold. (JPG 423 kb) Additional file 7: Adipophilin staining of coronary artery of familial hypercholesterolemia Bretoncelles Meishan (FBM) pig. Adipophilin staining of the right coronary artery of a FBM pig on atherogenic diet. A 2.5-fold showing classical asymmetric lesion formation. B Tenfold, C 20-fold and D 40-fold magnification. Adipophilin is a marker of lipid accumulation. Note the marked expression of adipophilin in the pathologically thickened intimal layer, whereas the unaffected intimal regions of the vessel are negative for adipophilin. (JPG 150 kb)
