# Erratum to: Left ventricular function during porcine-resuscitated septic shock with pre-existing atherosclerosis

**DOI:** 10.1186/s40635-016-0092-3

**Published:** 2016-06-28

**Authors:** Benedikt L. Nußbaum, Oscar McCook, Clair Hartmann, José Matallo, Martin Wepler, Elena Antonucci, Miriam Kalbitz, Markus Huber-Lang, Michael Georgieff, Enrico Calzia, Peter Radermacher, Sebastian Hafner

**Affiliations:** Klinik für Anästhesiologie, Universitätsklinik Ulm, Ulm, Germany; Institut für Anästhesiologische Pathophysiologie und Verfahrensentwicklung, Universitätsklinik Ulm, Helmholtzstraße 8/1, Ulm, 89081 Germany; Department of Surgical Sciences and Integrated Diagnostics, IRCCS San Martino IST, University of Genova, Genova, Italy; Klinik für Unfall-, Hand-, Plastische- und Wiederherstellungschirurgie, Universitätsklinik Ulm, Ulm, Germany

## Erratum

Unfortunately, after publication of this article [1], it was noticed that the ‘Statistical analysis’ section contained an error. The first sentence should read, “All data are expressed as median (IQ range)” to remain congruous with data displayed in tables. The original article has been updated to reflect this.
