# Supplementary material for: Left ventricular function during porcine-resuscitated septic shock with pre-existing atherosclerosis
Source: Intensive Care Med Exp. 2016 Jun 6;4:14. doi: 10.1186/s40635-016-0089-y (PMC4894859; doi:10.1186/s40635-016-0089-y)
Supplement: Additional file 1: — Phenotype of familial hypercholesterolemia Bretoncelles Meishan (FBM) pigs. Phenotype of familial hypercholesterolemia Bretoncelles Meishan (FBM) pigs with atherogenic diet for at least 9 months compared to healthy German landrace swine. n = 20 for FBM, n = 15 for landrace for cholesterol; n = 19 each for creatinine clearance, nitrite/nitrate and 8-isoprostane. Data are median (range) or mean ± standard deviation [14]. (DOCX 55 kb) [file 40635_2016_89_MOESM1_ESM.docx]

| **Additional file 1 Phenotype of familial hypercholesterolemia Bretoncelles Meishan (FBM) pigs** | | | |
| --- | --- | --- | --- |
|  | **FBM** | **Healthy Landrace** |  |
| Cholesterol (mmol/l) | 11.1 (7.4-12.3) | 1.4 (1.4-1.5) | p < 0.001 |
| Creatinine clearance (ml/min) | 72 ± 23 | 97 ± 26 | p = 0.004 |
| Nitrite/nitrate (µmol/l) | 14 ± 36 | 77 ± 80 | p < 0.001 |
| 8-isoprostane (pg/ml) | 111 ± 47 | 74 ± 16 | p = 0.005 |
| Phenotype of familial hypercholesterolemia Bretoncelles Meishan (FBM) pigs with atherogenic diet for at least nine months compared to healthy German Landrace swine. n=20 for FBM, n=15 for Landrace for cholesterol; n=19 each for creatinine clearance, nitrite/nitrate and 8-isoprostane. Data are median (range) or mean ± standard deviation. (Matějková et al. (2013) Intensive Care Med 39:497). | | | |
