# Supplementary material for: Left ventricular function during porcine-resuscitated septic shock with pre-existing atherosclerosis
Source: Intensive Care Med Exp. 2016 Jun 6;4:14. doi: 10.1186/s40635-016-0089-y (PMC4894859; doi:10.1186/s40635-016-0089-y)
Supplement: Additional file 2: — Response to ischemia/reperfusion injury of familial hypercholesterolemia Bretoncelles Meishan (FBM) pigs. Response to kidney ischemia/reperfusion injury with comparable post-ischemic organ dysfunction of familial hypercholesterolemia Bretoncelles Meishan (FBM) pigs with atherogenic diet for at least 9 months compared to healthy German landrace swine. n = 7 for FBM, n = 10 for landrace. Data are median (range) [14, 15]. (DOCX 14 kb) [file 40635_2016_89_MOESM2_ESM.docx]

| **Additional file 2: Table S2 Response to ischemia/reperfusion injury of familial hypercholesterolemia Bretoncelles Meishan (FBM) pigs** | | | | |
| --- | --- | --- | --- | --- |
|  |  | **pre-injury** | **8h post-injury** |  |
| Nitrite/nitrate (µmol/l) | FBM | 5 (1–9) | 19 (9–51) | p<0.05 |
|  | Landrace | 51 (11 – 141) | 48 (30 – 87) |  |
| 8-isoprostane (pg/ml) | FBM | 98 (77–233) | 280 (83–924) | p<0.05 |
|  | Landrace | 69 (62 – 130) | 78 (49 – 146) |  |
| Response to kidney ischemia/reperfusion injury with comparable post-ischemic organ dysfunction of familial hypercholesterolemia Bretoncelles Meishan (FBM) pigs with atherogenic diet for at least nine months compared to healthy German Landrace swine. n=7 for FBM, n=10 for Landrace. Data are median (range). (Matějková et al. (2013) Intensive Care Med 39:497 and Simon et al. (2011) Shock 35:156). | | | | |
