# Supplementary figures and images for: Left ventricular function during porcine-resuscitated septic shock with pre-existing atherosclerosis
Source: Intensive Care Med Exp. 2016 Jun 6;4:14. doi: 10.1186/s40635-016-0089-y (PMC4894859; doi:10.1186/s40635-016-0089-y)

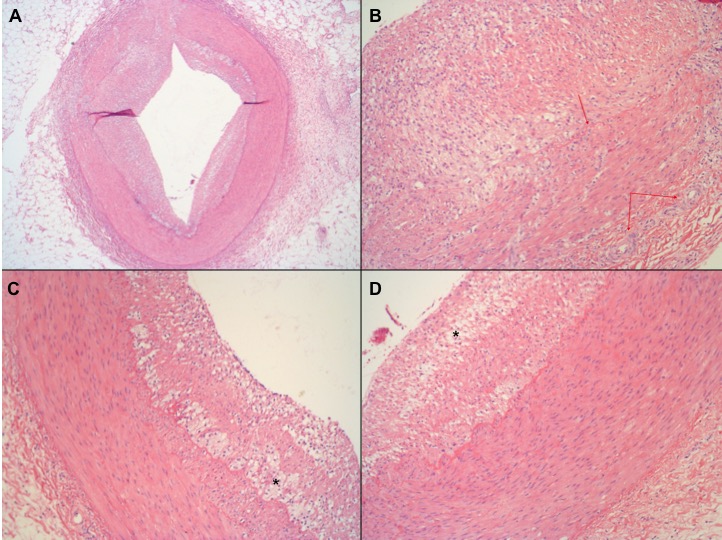

Supplement: Additional file 3: — Histology of coronary artery of familial hypercholesterolemia Bretoncelles Meishan (FBM) pig. Haematoxylin staining of the left coronary artery of a FBM pig on atherogenic diet. A. 2.5-fold magnification demonstrating pronounced pathological intimal thickening with narrowing of the arterial lumen. B–D. Tenfold magnification of different areas of the vessel. Asterisk (*) indicates areas of strong lipid accumulation. Red arrows mark the internal elastic lamina (single arrow) and the outer border of the media (double arrow). (JPG 140 kb) [file 40635_2016_89_MOESM3_ESM.jpg]

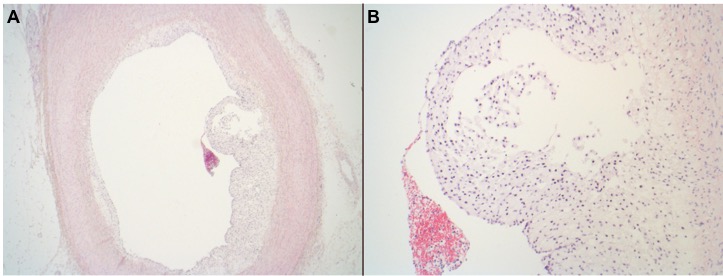

Supplement: Additional file 4: — Histology of coronary artery of familial hypercholesterolemia Bretoncelles Meishan (FBM) pig. Haematoxylin staining of coronary artery of a FBM pig on atherogenic diet. A 2.5- and B tenfold magnification demonstrating asymmetric atherosclerotic alterations with an instable plaque containing a large lipid core and associated thrombus formation. (JPG 52 kb) [file 40635_2016_89_MOESM4_ESM.jpg]

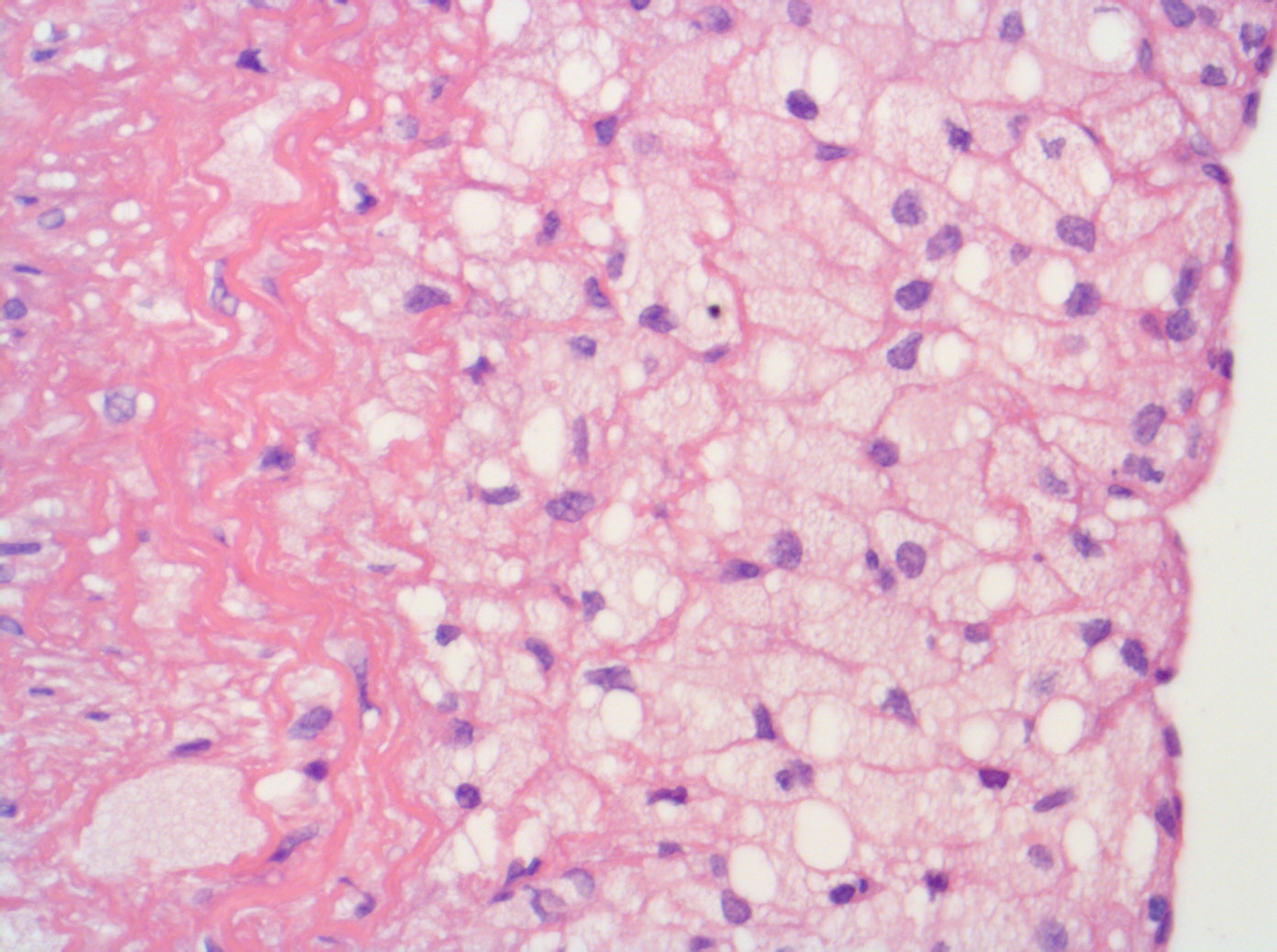

Supplement: Additional file 5: — Histology of coronary artery of familial hypercholesterolemia Bretoncelles Meishan (FBM) pig. Haematoxylin staining of right coronary artery of a FBM pig on atherogenic diet showing intimal thickening with marked lipid accumulation. ×40 magnification. (JPG 345 kb) [file 40635_2016_89_MOESM5_ESM.jpg]

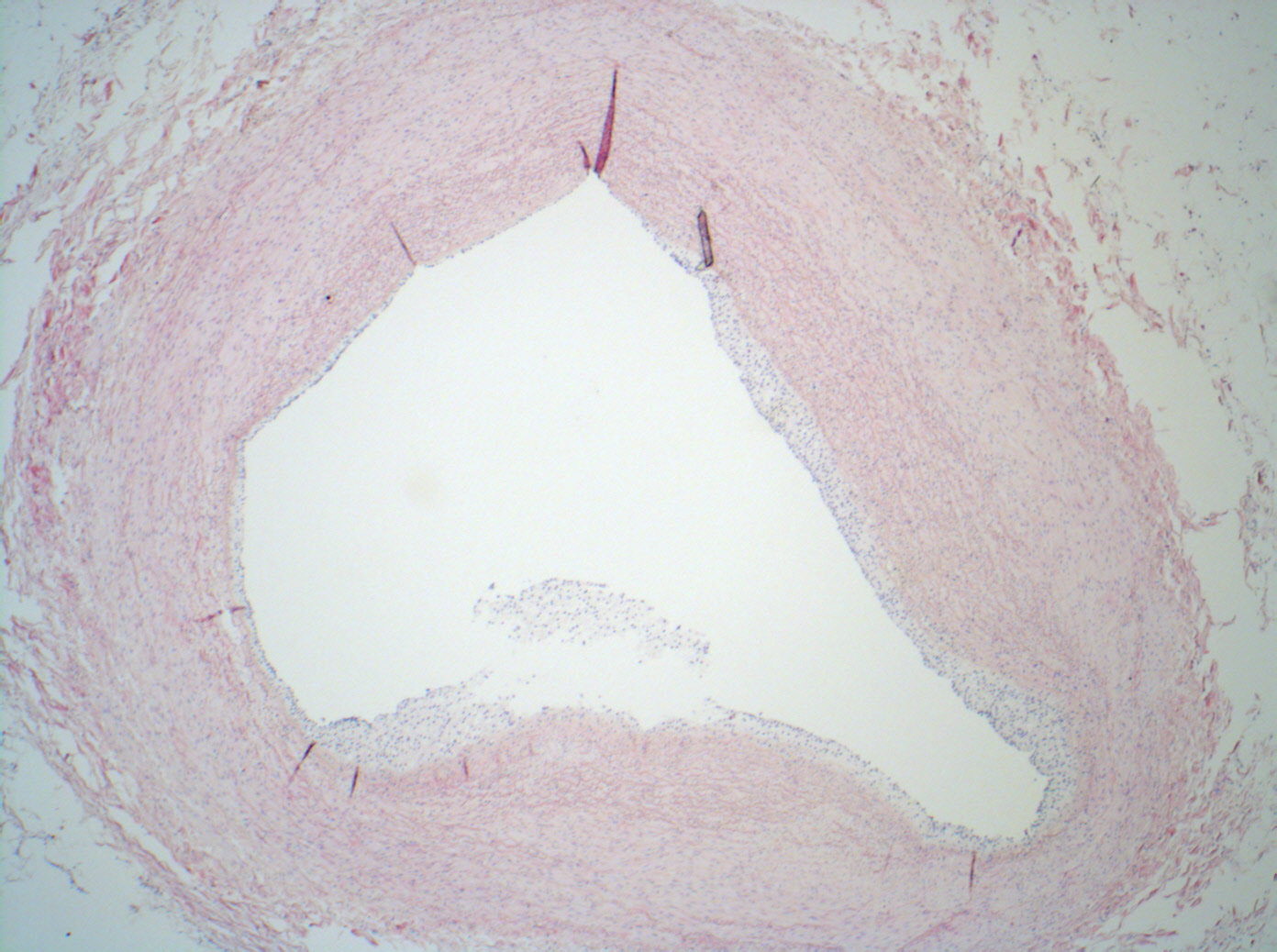

Supplement: Additional file 6: — Histology of coronary artery of familial hypercholesterolemia Bretoncelles Meishan (FBM) pig. Haematoxylin staining of coronary artery of a FBM pig on atherogenic diet demonstrating asymmetric atherosclerotic lesion formation with luminal narrowing. Note the coincident medial thickening at sites of intimal proliferation in contrast to unaffected regions of the vessel. Magnification of 2.5-fold. (JPG 423 kb) [file 40635_2016_89_MOESM6_ESM.jpg]

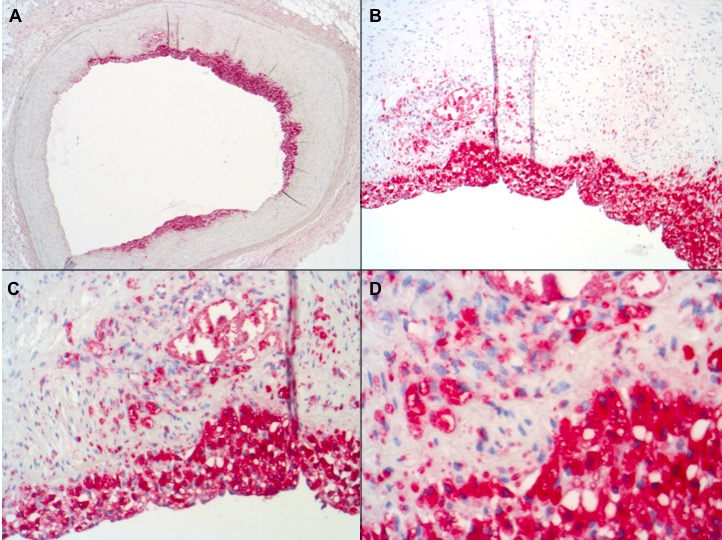

Supplement: Additional file 7: — Adipophilin staining of coronary artery of familial hypercholesterolemia Bretoncelles Meishan (FBM) pig. Adipophilin staining of the right coronary artery of a FBM pig on atherogenic diet. A 2.5-fold showing classical asymmetric lesion formation. B Tenfold, C 20-fold and D 40-fold magnification. Adipophilin is a marker of lipid accumulation. Note the marked expression of adipophilin in the pathologically thickened intimal layer, whereas the unaffected intimal regions of the vessel are negative for adipophilin. (JPG 150 kb) [file 40635_2016_89_MOESM7_ESM.jpg]
